# Rumor propagation dynamic analysis model based on hypergraph integration in public health events

**DOI:** 10.3389/fpubh.2026.1780116

**Published:** 2026-05-01

**Authors:** Mengna Zhang, Xin Zhang, Fuzhao Li

**Affiliations:** 1 School of Management Science and Engineering, Rural Revitalization Industry-Academia-Research-Application Center, Guizhou University of Finance and Economics, Guiyang, China; 2 College of Public Administration, Guizhou University of Finance and Economics, Guiyang, China; 3 Party and Government Office, Guizhou University of Finance and Economics, Guiyang, China

**Keywords:** public health emergencies, hypernetwork, rumor propagation, key dimensions, hesitant spreaders

## Abstract

During public health emergencies, the spread of online rumors can easily trigger public panic and anxiety, exacerbating their negative social impact. How to precisely regulate the dissemination of rumors in cyberspace under such circumstances, thereby enhancing the efficiency of public opinion management, has become a critical issue urgently needing resolution by both administrators and researchers. The study focuses on accurately describing the propagation patterns of rumor information from its source. Therefore, the study constructs a novel H-SNIR (Hypernetwork-Susceptible-Neglected-Infectious-Recovered) model in a hypernetwork. The model comprehensively considers the psychological differences among individuals during rumor propagation, categorizing rumor spreaders into two types: ordinary spreaders and hesitant spreaders. The H-SNIR model introduces a node for neglected spreaders, making the constructed model more consistent with the real-world rumor propagation process. Additionally, by integrating three key dimensions—user influence, topic popularity, and user interaction—the study thoroughly analyzes how these variables affect the rumor-forwarding mechanism in social networks.

## 1 Introduction

The complex network structure indicates that the spread of rumors in social networks is intricate and variable, and the opportunities for connections between different nodes are not equal. Factors such as the strength of connections between nodes, the influence of nodes, and the propagation environment all affect the spread of rumors in the network. Based on this, during public crisis events, to establish a rumor propagation model that better aligns with real-world dissemination patterns, it is necessary to explore the rumor propagation mechanism by considering both the network structure and the influencing factors of rumor propagation.

Traditional research on rumor propagation primarily relies on complex network modeling to analyze the propagation mechanisms of rumors in online social networks (1, 2). Complex networks consist of numerous “nodes” and some “edges” connecting two nodes, where network nodes can represent individuals in real social networks, and the edges between two nodes represent specific interactions between individuals (3). However, as the number of social network users increases, the relationships between users become more diverse. Hypernetwork theory provides an effective approach to describing complex relationships in real-world networks. Berge (4) first introduced the basic concepts and properties of hypergraphs. The core feature of hypergraphs is the introduction of “hyperedges,” which allow any number of homogeneous or heterogeneous nodes to be connected, reflecting existing relationships among multiple nodes. Li et al. (5) constructed a hypergraph-based SIS (Susceptible-Infectious-Susceptible) model for online rumor propagation, analyzing and simulating the global propagation process of rumors in online social networks. Yu P et al. (6) studied the topological properties and mechanisms of hypernetworks and proposed a hypernetwork evolution model based on hypergraphs, concluding that evolution models based on hypernetworks contribute to understanding the structure and evolution mechanisms of real social networks.

In recent years, many scholars have conducted in-depth explorations and researches on the rumor spreading mechanism. Due to the large number of users in social networks, the traditional SEIR model can no longer fully express the state of all users in social networks. With scholars' in-depth exploration and research on rumor spreading, many scholars have incorporated hesitation mechanism, education level, and the counter-attack mechanism proposed by early psychologists into the rumor spreading model. Zhao et al. (7) introduced memory and forgetting mechanisms into isomorphic networks and established the SHIR model to study the effects of spreading rate, forgetting rate and average network degree on rumor spreading. They found that the forgetting and remembering mechanisms present in hibernators delayed the end of the rumor and reduced its maximum impact to some extent. Hu et al. (8) examined the effect of changes in the proportion of wise people in a population on the spread of rumors. They demonstrated that an increase in the proportion of wise people in the population has a systematic effect on solving the problem of rumor spreading. In addition, the role of social media in the spread of rumors cannot be ignored. Li et al. (9) developed an augmented rumor spreading model with a special focus on the impact of knowledge education and intervention strategies on reducing rumor spreading. Their results suggest that enhanced education on rumor identification and timely disinformation are very effective in controlling rumor spread. Xia et al. (10) proposed the SEIR model by adding the new factor of hesitation mechanism to the classical SIR model. Hong et al. (11) added the factor of investor network reality to the SEIR model, and then proposed the SE2IR rumor spreading model with a hesitation mechanism. Therefore, based on the existing literature, this study provides a more comprehensive analysis of the rumor spreading mechanism.

Based on the above analysis, this study establishes a new model of rumor dissemination. The model takes into account the role of the rumor-busting individual in guiding other individuals in different states, which we refer to as the anti-rumor mechanism. Meanwhile, this study quantitatively calculates the state transfer probability of the rumor propagation model based on the effects of different influencing factors on the rumor propagation mechanism. By quantitatively calculating the influencing factors, it is possible to more precisely dissect the spreading characteristics of social network rumors at different stages. In order to more accurately calculate the influence of different influencing factors on the spread of rumor information, this study combines the microscopic Markov (MMC) theory to accurately calculate the transfer probability of nodes between different states, so as to accurately express the influencing factors and state transfer probability between susceptible, latent, infected, and immune nodes in a complex network.

The innovations of our study are as follows:

(1) A novel H-SNIR rumor propagation model integrating a neglected mechanism within a super-network is constructed to deeply analyze the impact of topic popularity, user influence, and user interaction on rumor information forwarding behavior.
(2) By quantitatively representing these three-dimensional influencing factors, the model quantitatively evaluates the contribution of different factors to online rumor propagation during sudden public crisis events. It transforms the fixed transition probabilities between nodes into computable dynamic parameters.
(3) A Markov chain dynamic equation is established to accurately calculate the transition probabilities of nodes between different states based on the influencing factors, abstracting the rumor information propagation process into a probabilistic system of state transitions, thereby more realistically simulating the complex and non-steady-state propagation behaviors in real-world social networks.

## 2 Method

### 2.1 H-SNIR propagation model

The topological structure of networks significantly influences the spread of rumor information (12). Particularly when studying the mechanisms of rumor dissemination in social networks, the impact of network structure cannot be overlooked. Current research on the mechanisms of rumor information propagation is largely based on complex networks such as random networks (13, 14), small-world networks (15), and scale-free networks (16). However, in the actual process of rumor dissemination, complex networks often fail to accurately describe the topological structure of social networks (17). For instance, scale-free networks can better describe the “rich get richer” node degree distribution characteristic, which aligns with the super-spreader effect of key opinion leaders. Yet, as an increasing number of strangers connect through convenient online platforms to form network communities based on shared interests, topics, or needs, scale-free networks cannot fully capture the topological structure of virtual communities (18, 19). Hypergraphs more accurately depict the group communication behaviors involving multiple users in real-world social networks. Research on information dissemination mechanisms based on hypergraphs has already garnered attention from scholars (20–22).

#### 2.1.1 The hypergraph theory

Graphs are a classic method for representing social networks. In traditional social networks, nodes represent users, while edges denote binary relationships between pairs of users. However, interactions in real-world social networks often occur among multiple users. The conventional graph structure can only capture pairwise interactions and fails to fully characterize such higher-order relationships. Berge argued that hypergraphs overcome the limitations of traditional graph models in representing binary relations by capturing interactions among multiple nodes.

Assuming *V* = {*v*_1_, *v*_2_, *v*_3_, ⋯ , *v*_*n*_} represents the set of nodes, *E* = {*e*_1_, *e*_2_, *e*_3_, ⋯ , *e*_*m*_} represents the set of hyperedges, we define *H* = (*V, E*) as a hypergraph. If |*e*_*i*_| = *k*(*i* = 1, 2, 3, ⋯ , *m*), then *H* = (*V, E*) is called a k-uniform hypergraph. If |*e*_*i*_| = 2, then *H* = (*V, E*) degenerates into an ordinary graph in complex network studies.

**Figure 1:**
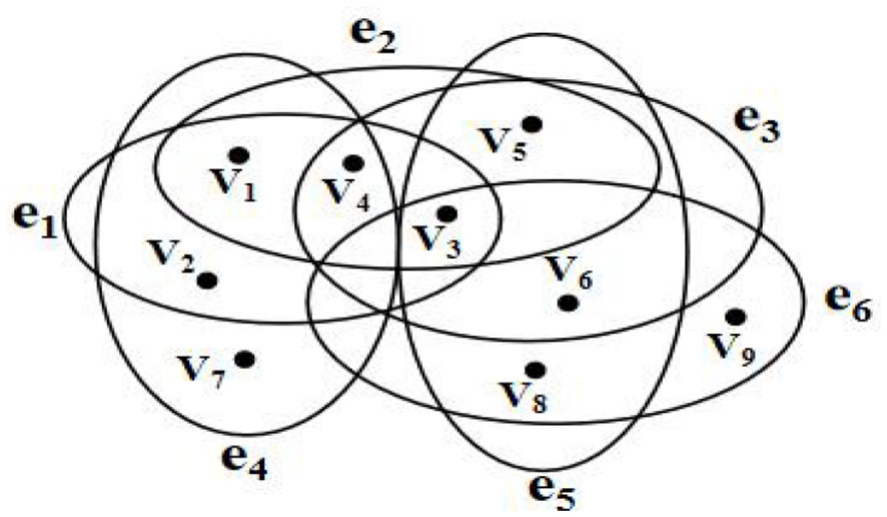
Schematic diagram of a 4-uniform hypernetwork.

The schematic diagram of a hypergraph is shown in Figure 1. In this hypernetwork, *V* = {*v*_1_, *v*_2_, *v*_3_, *v*_4_, *v*_5_, *v*_6_, *v*_7_, *v*_8_, *v*_9_} represents the set of nodes, *E* = {*e*_1_, *e*_2_, *e*_3_, *e*_4_, *e*_5_, *e*_6_} represents the set of hyperedges. If nodes *v*_1_ and *v*_2_ belong to the same hyperedge *e*_1_, then they are considered adjacent nodes *v*_1_, *v*_2_. If hyperedges *e*_1_ and *e*_4_ share common nodes *v*_1_, *v*_2_, *v*_4_, then *e*_1_, *e*_4_ are considered adjacent hyperedges. |*e*_*i*_| = 4. As shown in Figure 1, this is a 4-uniform hypernetwork. As shown in Figure 1, this is a 4-uniform hypernetwork.

#### 2.1.2 The construction process of the hypernetwork

Our study improves the BA scale-free hypernetwork model as a foundation to ultimately construct a hypernetwork. As the underlying topological structure of the model, the hypernetwork is constructed through the following process.

**Figure 2:**
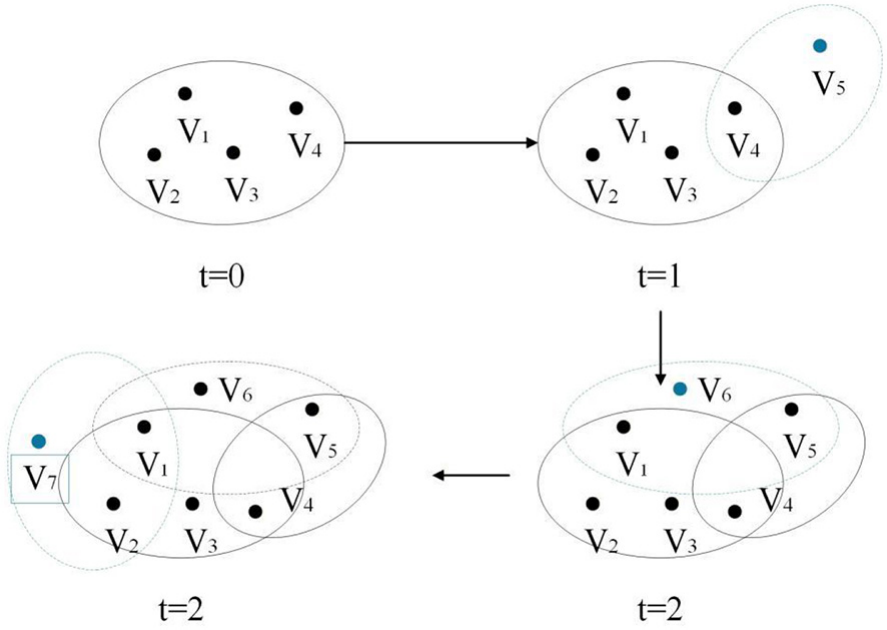
Schematic diagram of the hypernetwork evolution process.

(1) Initialization: At the initial moment, the hypernetwork consists of m_0_ user nodes and one topic node forming a hyperedge, it indicates that the initial users participate in the discussion of a certain topic, thereby it formes the initial social group.
(2) Growth: At each time step, new user nodes or topic nodes are added to the network to simulate the process of users continuously joining the platform and initiating new topics. In social networks, users are more inclined to participate in discussions on topics rather than proactively initiating topics. During the node addition process, hyperedges are generated in the following two ways: If the added node is a user node, a certain number of *m*_1_ existing topic nodes are selected, and the new user node is preferentially connected to topic nodes with higher participation levels, topics that are more frequently discussed. If the added node is a topic node, a certain number of existing user nodes *m*_2_ are randomly selected, and the new topic node is preferentially connected to active users, thereby simulating the scenario where a new topic is first engaged by active users. Figure 2 is an example diagram illustrating the evolution process of a hypernetwork. In the diagram, black nodes represent existing users or topics, blue nodes represent newly added users or topics, and dotted curves represent newly formed participation relationships in discussions.

#### 2.1.3 H-SNIR model equations

**Figure 3:**
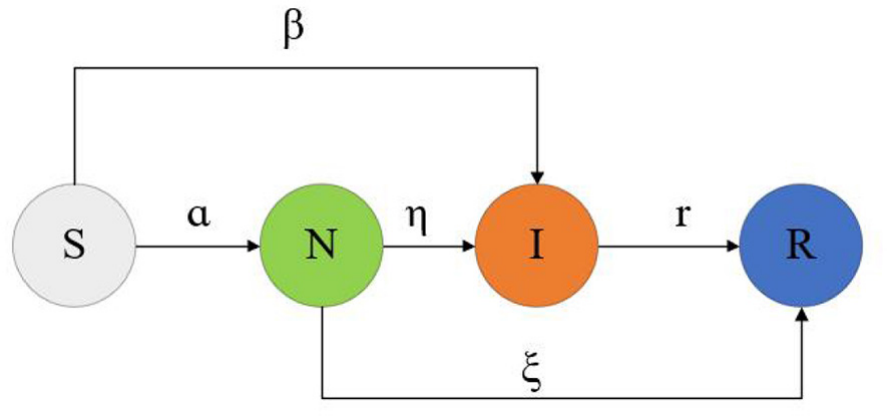
State transition diagram of the H-SNIR model in a hypernetwork.

Epidemic dynamics models employ mathematical methods to simulate and analyze the trends of infectious disease transmission. Building upon the SEIR epidemic dynamics model, this chapter introduces a hesitant node (Neglected, N) based on the characteristics of rumor propagation in public crisis events, thereby constructing an H-SNIR model for rumor propagation based on hypernetworks. In social networks, not all nodes choose to spread rumors upon receiving them. Some nodes may hesitate to decide whether to propagate the rumor due to uncertainty about the authenticity of the information. Accordingly, user nodes are categorized into four states: Susceptible (S), Neglected (N), Infectious (I), and Recovered (R). The state transitions of various node types in the H-SNIR model are illustrated in Figure 3.

(1) Susceptible nodes (S) represent those who are completely unaware of the rumor.
(2) Neglected nodes (N) represent those who are hesitant and undecided about whether to spread the rumor.
(3) Infectious nodes (I) represent those who believe in and actively spread the rumor.
(4) Recovered nodes (R) represent those who have stopped spreading the rumor.

The transition rules between users of different states in the H-SNIR model are defined as follows:

(1) In the initial hypernetwork, there are I_0_ nodes in the infected state, while all other nodes are in the susceptible state.
(2) At each time step, infected nodes in the hypernetwork spread the rumor to all neighbor nodes within the same hyperedge. Influenced by factors such as user influence, topic popularity, and interactions between users, susceptible nodes S become aware of the rumor with probability α but, due to uncertainty about its authenticity, transition to the hesitant state N or with probability β, they become aware of the rumor, believe it, and transition to the infected state I.
(3) Hesitant nodes N, repeatedly exposed to the rumor, may believe and forward it with probability η, transitioning to the infected state I. Meanwhile, some hesitant nodes N, affected by factors such as forgetfulness or loss of interest in the rumor, stop spreading it and transition to the recovered state R with probability ξ.
(4) Over time, infected nodes I, influenced by forgetfulness or loss of interest in the rumor, cease spreading it and transition to the recovered state R with probability γ.
(5) During the rumor propagation process, the final state of nodes is the recovered state R. Nodes in the recovered state no longer undergo changes. As the rumor continues to spread through the hypernetwork, the densities of nodes in each state reach a stable value, fluctuating slightly around it, indicating that the rumor propagation state in the hypernetwork has reached a stable equilibrium.antitative representation of user and information attributes.

Based on the propagation rules described above, the differential equations for the dynamics of the H-SNIR model are given by Equation 1:


$$\begin{array}{l} \{\begin{array}{l} \frac{dS(t)}{dt}=-\alpha S(t)N(t)-\beta S(t)I(t)\\ \frac{dN(t)}{dt}=\alpha S(t)N(t)-\eta N(t)I(t)-\xi N(t)R(t)\\ \frac{dI(t)}{dt}=\eta I(t)N(t)+\beta S(t)I(t)-\gamma I(t)R(t)\\ \frac{dR(t)}{dt}=\xi N(t)R(t)+\gamma I(t)R(t) \end{array} \end{array}$$ (1)

The constraints for each parameter in Equation Set (1) are given by Equation 2:


$$\begin{array}{l} \{\begin{array}{l} S(t)+N(t)+I(t)+R(t)=1\\ \alpha +\beta =1,\alpha >0,\beta >0\\ \eta +\xi =1,\eta >0,\xi >0\\ \gamma >0 \end{array} \end{array}$$ (2)

**Figure 4:**
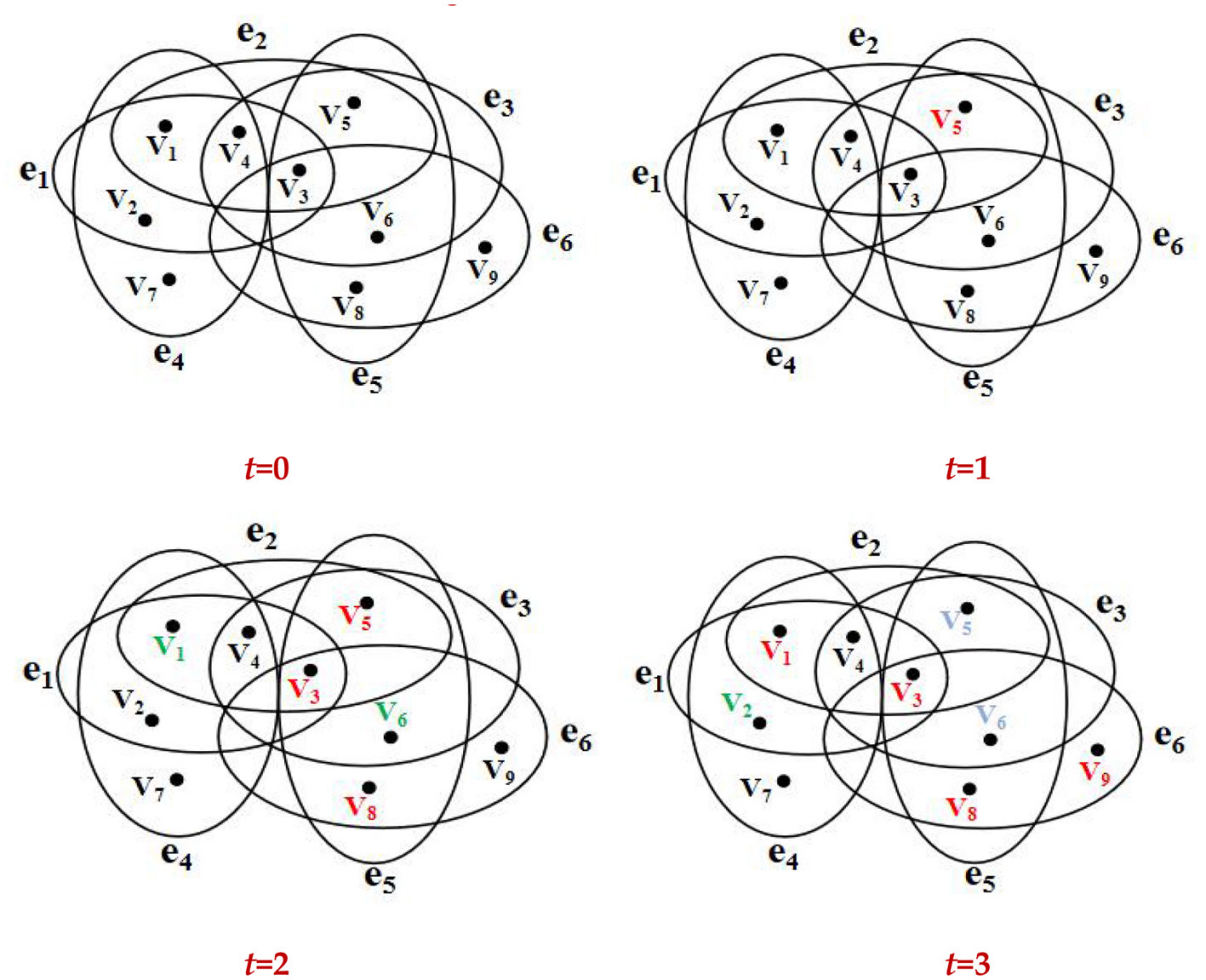
Schematic diagram of information propagation in an online social hypernetwork.

The propagation process of rumor information in an online social hypernetwork is illustrated in Figure 4. Here, black nodes, green nodes, red nodes, and blue nodes represent users in the state S, state N, state I, and state R. *t* = 0, all nodes in the hypernetwork are in the S state. *t* = 1, a node is randomly selected from the hypernetwork and set to the I state. Assume the randomly chosen node is numbered v_5_ and is marked in red. *t* = 2, since node *v*_5_ is surrounded by hyperedges *e*_2_*, e*_3_, and *e*_5_, its adjacent nodes *v*_1_ and *v*_6_ receive the rumor information but cannot decide whether to forward it, transitioning to the N state. Nodes *v*_3_and *v*_8_ receive the information and spread the rumor, transitioning to the I state. *t* = 3, node v_1_ transitions from the N state to the I state, while node v_6_ transitions from the N state to the R state. As nodes *v*_3_*, v*_5_ and *v*_8_ are surrounded by hyperedges *e*_1_*, e*_2_*, e*_3_*, e*_5_, and *e*_6_, the adjacent node v_2_ receives the information but does not spread the rumor, transitioning to the N state. Node *v*_9_ receives the information and enters the I state. Node *v*_5_ loses interest or the ability to spread the information, transitioning to the R state.

## 3 Theoretical analysis based on Markov chain methods

### 3.1 Quantitative representation of user and information attributes

#### 3.1.1 Definition 1. User influence

In a hypernetwork, users with high influence have more followers and are also more willing to forward rumor information. Therefore, the influence of user α(*P*^*i*^) is calculated as shown in Equation 3:


$$\begin{array}{l} \alpha \left(P^{i}\right)=m\cdot fansNum\left(v_{i}\right)+n\cdot \overset{k}{\underset{o=0}{\sum }}retNum\left(v_{i},v_{o}\right) \end{array}$$ (3)

In this equation, *fansNum* (*v*_*i*_) represents the number of followers of user $v_{i}.\overset{k}{\underset{o=0}{\sum }}retNum\left(v_{i},v_{o}\right)$ denotes total volume of rumors forwarded by user *v*_*i*_ to user *v*_0_, with *o* = 1, 2, ⋯ , *k* representing all users in the soal media platform. Here, *m* and *n* are weighting coefficients. Assuming that the number of followers and volume of rumor forwarding contribute equally to user influence, we set *m* = 0.5 and *n* = 0.5.

#### 3.1.2 Definition 2. Topic popularity

The betweenness centrality in a hypernetwork reflects the importance of a node as a “bridge.” The betweenness centrality *BC*_*v*_*i*__ of node *v*_*i*_ is calculated as shown in Equation 4.


$$\begin{array}{l} BC_{v_{i}}=\underset{p\neq q\neq v_{i}\in V^{\prime }}{\sum }\frac{\delta_{p,q}\left(v_{i}\right)}{\delta_{p,q}} \end{array}$$ (4)

In this equation, δ_*p, q*_(*v*_*i*_) represents the total number of shortest paths passing through node *v*_*i*_ among all pairs of nodes *p*→*q*, and δ_*p, q*_ denotes the total number of shortest paths between nodes *p*→*q*.

A new node is defined as one that has never previously appeared in the local network. The calculation of topic popularity is not simply based on counting the number of newly added nodes in the local network. Instead, weight is assigned to new nodes according to their betweenness centrality, which better measures the amount of non-redundant information these new nodes bring to the research topic. The popularity of a topic is calculated as shown in Equation 5, where *n*_*v*_*i*__ represents the new nodes that have emerged in the local network.


$$\begin{array}{l} n_{v_{i}}=\underset{v_{j}\in Vneigh}{\sum }BC_{v_{j}} \end{array}$$ (5)

In this equation, *BC*_*v*_*j*__ denotes the betweenness centrality of neighbor node, *V*_*nsigh*_ represents the set of neighbor nodes of node *v*_*i*_, *v*_*j*_ refers to the neighbor nodes of node *v*_*i*_ in the network, $\underset{v_{j}\in V_{neigh}}{\sum }BC_{v_{j}}$ represents the sum of the *v*_*i*_ betweenness centrality values *BC*_*v*_ of all neighbor nodes of node.

#### 3.1.3 Definition 3. Interactions between users

If node *j* is a neighbor of node *i* in the hypernetwork, the influence of node *j* on node *i* is defined as shown in Equation 6:


$$\begin{array}{l} f\left(i,j\right)=\frac{k_{j}}{\underset{l\in \Gamma \left(j\right)}{\sum }k_{l}} \end{array}$$ (6)

In this equation, *k*_*j*_ represents the degree of node j, and Γ(*j*)denotes the set of neighbor nodes of node *j*. The term $\underset{l\in \Gamma \left(j\right)}{\sum }k_{l}$ represents the sum of the degrees of all nodes *j* in the neighbor set Γ(*j*) of node *l*.

If node j is a neighbor of node i in the hypernetwork, the relative influence of node *j* on node *i* is defined as shown in Equation 7:


$$\begin{array}{l} rf\left(i,j\right)=\frac{2f\left(i,j\right)}{f\left(i,j\right)+f\left(j,i\right)} \end{array}$$ (7)

In this equation, *f(i,j)* represents the influence of node *j* on its neighbor node *i*, while *f(j,i)* denotes the influence of node *i* on its neighbor node *j*.

### 3.2 Markov chain dynamic equations

In traditional models of rumor spreading, a susceptible node *i* transitions to the infected state with a fixed probability, without considering the network influence of the spreader *j* or the mutual influence between users. However, this does not align with the characteristics of rumor propagation in real-world scenarios.

#### 3.2.1 Definition 4

Based on user influence, topic popularity, and interactions among users, this chapter defines a nonlinear comprehensive propagation rate as shown in Equation 8.


$$\begin{array}{l} \omega_{ij}^{I}\left(P^{I}\right)=\{\begin{array}{r} \omega \alpha \left(P^{I}\right)\cdot n_{i_{i}}\cdot rf(i,j),\;当\;\omega \alpha \left(P^{I}\right)\cdot n_{v_{i}}\cdot rf(i,j)<1\\ 1,\;当\;\omega \alpha \left(P^{I}\right)\cdot n_{v_{i}}\cdot rf(i,j)\geq 1 \end{array} \end{array}$$ (8)

In this equation, ω represents the baseline propagation rate of rumor information, α(*P*^*I*^) denotes user influence, *n*_*v*_*i*__ indicates topic popularity, and *rf*(*i, j*) captures the interactions between users.

The study employs the Markov chain method (23) to represent the dynamic propagation equations of the H-SNIR model. The probability that node *j* is not infected by node *i* at time *t* is defined *r*_*j*_(*t*) as shown in Equation 9:


$$\begin{array}{l} r_{j}\left(t\right)=\underset{k\neq j}{\prod }\left[1-\omega_{kj}^{I}\left(P^{I}\right)P_{k}^{I}\left(t\right)\right] \end{array}$$ (9)

In this equation, $\omega_{kj}^{I}\left(P^{I}\right)$ represents the nonlinear comprehensive propagation rate, $P_{k}^{I}\left(t\right)$ denotes the probability that node *k* is in state *I* at time *t*. Additionally, let $P_{k}^{S}\left(t\right),P_{k}^{N}\left(t\right),P_{k}^{I}\left(t\right)$, and $P_{k}^{R}\left(t\right)$ represent the probabilities that node *k* is in states S, N, I, and R at time *t*. These state probabilities satisfy the normalization condition: $P_{k}^{S}\left(t\right)+P_{k}^{N}\left(t\right)+P_{k}^{I}\left(t\right)+P_{k}^{R}\left(t\right)=1$.

According to the transition rules between users of different states in the H-SNIR model, the state transition probability of node *i* in state S at time *t* is given by Equation 10:


$$\begin{array}{l} \{\begin{array}{l} \theta_{i}^{s\to s}(t)=r_{i}^{N}(t)r_{i}^{I}(t)\\ \theta_{i}^{s\to N}(t)=\left[1-r_{i}^{N}(t)\right]r_{i}^{I}(t)\\ \theta_{i}^{s\to I}(t)=\left[1-r_{i}^{I}(t)\right]r_{i}^{N}(t)+\left[1-r_{i}^{N}(t)\right]\left[1-r_{i}^{I}(t)\right] \end{array} \end{array}$$ (10)

In this equation, $\theta_{i}^{S\to S}\left(t\right)+\theta_{i}^{S\to N}\left(t\right)+\theta_{i}^{S\to I}\left(t\right)=1,r_{i}^{N}\left(t\right)$ represents the probability that node *i* does not transition to the hesitant state N, and $r_{i}^{I}\left(t\right)$ denotes the probability that node *i* does not transition to the infected state I.

The state transition probability of node *i* in state N at time *t* is given by Equation 11:


$$\begin{array}{l} \{\begin{array}{l} \theta_{i}^{N\to N}(t)=r_{i}^{I}(t)r_{i}^{R}(t)\\ \theta_{i}^{N\to I}(t)=\left[1-r_{i}^{I}(t)\right]r_{i}^{R}(t)\\ \theta_{i}^{N\to R}(t)=\left[1-r_{i}^{R}(t)\right]r_{i}^{I}(t)+\left[1-r_{i}^{I}(t)\right]\left[1-r_{i}^{R}(t)\right] \end{array} \end{array}$$ (11)

In this equation, $\theta_{i}^{\text{N}\to \text{N}}\left(t\right)+\theta_{i}^{\text{N}\to I}\left(t\right)+\theta_{i}^{\text{N}\to R}\left(t\right)=1,r_{i}^{I}\left(t\right)$ represents the probability that node *i* does not transition to the infected state I, and $r_{i}^{R}\left(t\right)$ denotes the probability that node *i* does not transition to the recovered state *R*.

The state transition probability of node *i* in state I at time *t* is given by Equation 12:


$$\begin{array}{l} \{\begin{array}{l} \theta_{i}^{I\to I}(t)=1-r\\ \theta_{i}^{I\to R}(t)=r \end{array} \end{array}$$ (12)

In this equation, $\theta_{i}^{I\to I}\left(t\right)+\theta_{i}^{I\to R}\left(t\right)=1,r$ represents the probability that node *i* transition to the recovered state R.

The state transition probability of node *i* in state R at time *t* is given by Equation 13:


$$\begin{array}{l} \theta_{i}^{R\to R}\left(t\right)=1 \end{array}$$ (13)

**Figure 5:**
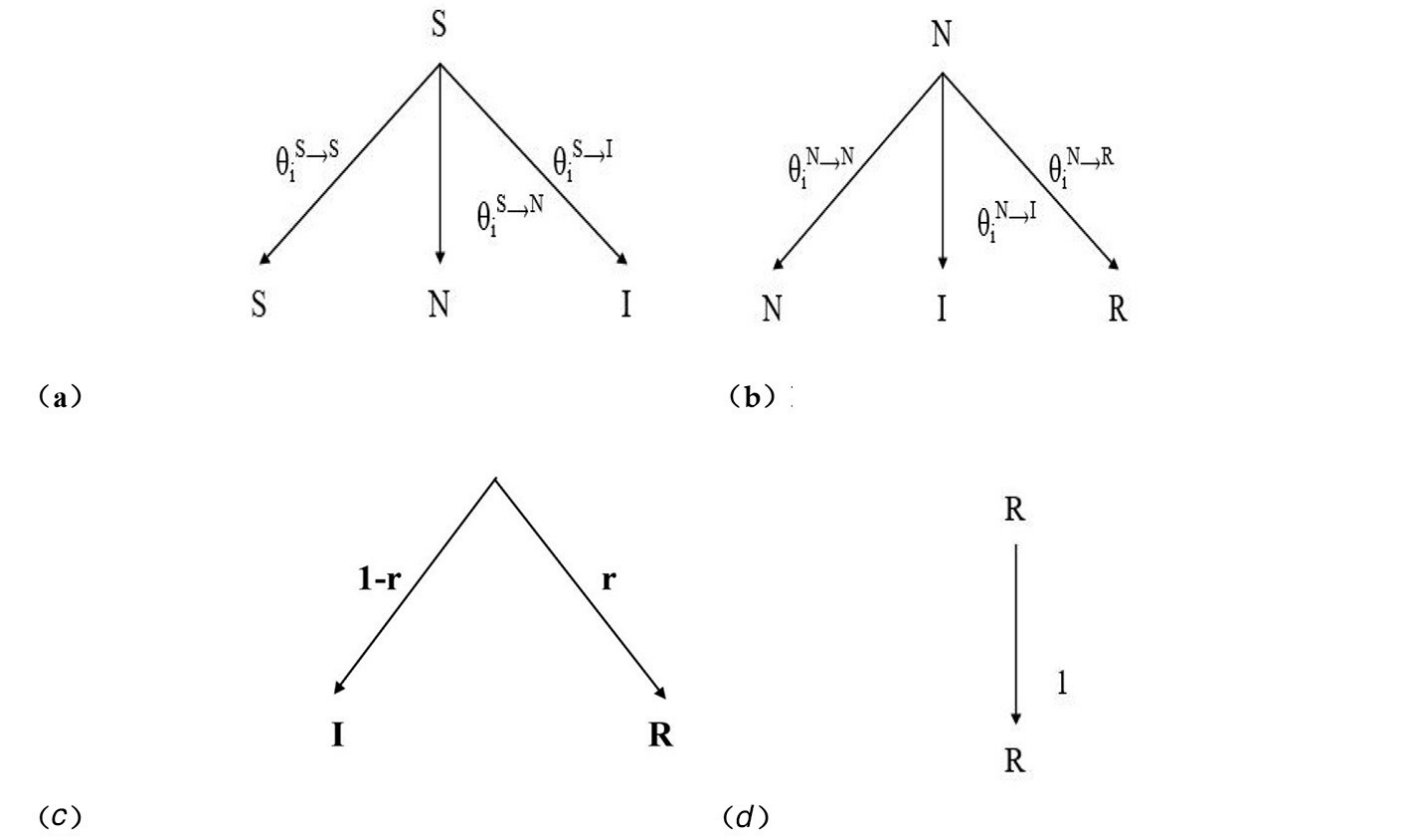
Probability transfer trees for the four states. **(a)** Probability transfer tree with initial state S, **(b)** Probability transfer tree with initial state N. **(a)** Probability transfer tree with initial state I, **(b)** Probability transfer tree with initial state R.


Based on the calculation of the state transition probabilities above, the probability transfer trees for the four states (S, N, I, R) in the rumor propagation system of this paper can be derived, as shown in Figure 5. The root node of each probability transfer tree represents the state of node ii at the current time step, while the leaf nodes represent the possible states that node ii may transition to at the next time step. Therefore, based on the Markov chain method, the dynamic equations of the H-SNIR model can be expressed as Equation 14:

$$\begin{array}{l} \{\begin{array}{l} P_{i}^{S}(t+1)=P_{i}^{S}(t)\cdot \theta_{i}^{S\to S}(t)\\ P_{i}^{N}(t+1)=P_{i}^{S}(t)\cdot \theta_{i}^{S\to N}(t)+P_{i}^{N}(t)\theta_{i}^{N\to N}(t)\\ P_{i}^{I}(t+1)=P_{i}^{S}(t)\theta_{i}^{S\to I}(t)+P_{i}^{N}(t)\cdot \theta_{i}^{N\to I}(t)+P_{i}^{I}(t)(1-r)\\ P_{i}^{R}(t+1)=P_{i}^{N}(t)\theta_{i}^{N\to R}(t)+P_{i}^{I}(t)\cdot r+P_{i}^{R}(t)\theta_{i}^{R\to R}(t) \end{array} \end{array}$$ (14)

In the equation, $P_{i}^{S}\left(t+1\right)$ represents the probability of being in the susceptible state *S* at time *t*+1, $P_{i}^{S}\left(t\right)$ denotes the probability of being in the susceptible state S at time *t*, and $\theta_{i}^{S\to S}\left(t\right)$ indicates the transition probability of node *i* from state S to state S at time *t*. The other terms follow a similar representation.

### 3.3 Threshold for rumor outbreak

The study analyzes the propagation threshold of rumor information I in social networks based on the dynamic equations of the H-SNIR model. When the propagation rate of rumor information I is below its outbreak threshold, the rumor will fade out in the social network. Conversely, when the propagation rate exceeds the threshold, the information will persist and not disappear.

**Theorem 1**: If only rumor information I exists in the social network, the propagation threshold of rumor information I is given by Equation 15:


$$\begin{array}{l} \omega^{I}=\frac{\mu }{\Lambda_{\max}\left(H\right)\cdot \alpha \left(P^{I}\right)\cdot n_{v_{i}}} \end{array}$$ (15)

In this equation, Λ_*max*_(*H*) represents the largest eigenvalue of matrix H, where the elements of matrix H are given by *h*_*ij*_ = *c*_*ij*_*rf*(*i, j*).

Proof: When the propagation of rumors in a social network reaches a stable state, the transition probabilities of node *i* in states S, N, and I no longer change over time. Therefore, assume the probability of being in state S at time *t*+1 is $P_{i}^{s}\left(t+1\right)=P_{i}^{s}\left(t\right)=P_{i}^{s}$, the probability of being in state N at time *t*+1 is $P_{i}^{N}\left(t+1\right)=P_{i}^{N}\left(t\right)=P_{i}^{N}$, the probability of being in state I at time t+1 is $P_{i}^{I}\left(t+1\right)=P_{i}^{I}\left(t\right)=P_{i}^{I}$, and the transition probability from state S to state I at time *t* is $\theta_{i}^{s\to I}\left(t\right)=\theta_{i}^{s\to I}$, the transition probability from state N to state I at time *t* is $\theta_{i}^{N\to I}\left(t\right)=\theta_{i}^{N\to I}$, and the transition probability from state I to state I at time *t* is $\theta_{i}^{I\to I}\left(t\right)=\theta_{i}^{I\to I}$. Then, the probability of being in state *I* for node *i* is calculated as shown in Equation 16:


$$\begin{array}{l} \begin{array}{c} P_{i}^{I}=P_{i}^{S}\theta_{i}^{S\to I}+P_{i}^{N}\theta_{i}^{N\to I}+P_{i}^{I}(1-r)\\ =P_{i}^{S}\left[1-r_{i}^{I}(t)\right]r_{i}^{N}(t)+P_{i}^{N}\left[1-r_{i}^{I}(t)\right]r_{i}^{R}(t)+P_{i}^{I}(1-r) \end{array} \end{array}$$ (16)

The transition probabilities for nodes S, N, and R are calculated as shown in Equation 17:


$$\begin{array}{l} \{\begin{array}{c} P_{i}^{S}=P_{i}^{S}\cdot \theta_{i}^{S\to S}=P_{i}^{S}\cdot r_{i}^{N}\cdot r_{i}^{I}\\ P_{i}^{N}=P_{i}^{S}\cdot \theta_{i}^{S\to N}+P_{i}^{N}\cdot \theta_{i}^{N\to N}=P_{i}^{S}\cdot \left(1-r_{i}^{N}\right)r_{i}^{I}+P_{i}^{N}\cdot r_{i}^{I}r_{i}^{R}\\ P_{i}^{R}=P_{i}^{N}\cdot \theta_{i}^{N\to R}+P_{i}^{I}\cdot r+P_{i}^{R}\\ =P_{i}^{N}\cdot \left[\left(1-r_{i}^{R}\right)r_{i}^{I}+\left(1-r_{i}^{I}\right)\left(1-r_{i}^{R}\right)\right]+P_{i}^{I}\cdot r+P_{i}^{R} \end{array} \end{array}$$ (17)

During the rumor propagation process, the number of infected nodes I and hesitant nodes N in the stable state is extremely low. Therefore, it is assumed that $P_{i}^{I}=\varepsilon_{i}\ll 1,P_{i}^{N}=\varepsilon_{i}\ll 1$, and the corresponding $r_{i}^{S},r_{i}^{N},r_{i}^{I},r_{i}^{R}$ can be approximated as shown in Equation 18:


$$\begin{array}{l} \{\begin{array}{l} r_{i}^{S}\approx 1-\tau_{i}^{S}\\ r_{i}^{N}\approx 1-\tau_{i}^{N}\\ r_{i}^{I}\approx 1-\tau_{i}^{I}\\ r_{i}^{R}\approx 1-\tau_{i}^{R} \end{array} \end{array}$$ (18)

In the formula, $\tau_{i}^{s}=\omega \alpha \left(P^{s}\right)\cdot n_{v_{i}}\cdot \underset{j}{\sum }rf\left(i_{i},j\right)P_{ij}^{s}\left(t\right),\tau_{i}^{N}=\omega \alpha \left(P^{N}\right)\cdot n_{v_{i}}\cdot \underset{j}{\sum }rf\left(i_{i},j\right)P_{ij}^{N}\left(t\right)$, $\tau_{i}^{I}=\omega \alpha \left(P^{I}\right)\cdot n_{v_{i}}\cdot \underset{j}{\sum }rf\left(i_{i},j\right)P_{ij}^{I}\left(t\right),\tau_{i}^{R}=\omega \alpha \left(P^{R}\right)\cdot n_{v_{i}}\cdot \underset{j}{\sum }rf\left(i_{i},j\right)P_{ij}^{R}\left(t\right)$.

Based on Equations 17, 18, the dynamic equations of the social network in a steady state can be derived, as shown in Equation 19:


$$\begin{array}{l} \{\begin{array}{l} P_{i}^{S}=P_{i}^{S}\left(1-\tau_{i}^{N}\right)r\\ P_{i}^{N}=P_{i}^{S}\tau_{i}^{N}r+P_{i}^{N}r(1-r)\\ P_{i}^{R}=P_{i}^{N}r+P_{i}^{I}r+P_{i}^{R} \end{array} \end{array}$$ (19)

Substituting Equation 18 into Equation 16 yields a new equation, as shown in Equation 20:


$$\begin{array}{l} P_{i}^{I}=P_{i}^{S}\tau_{i}^{I}\left(1-\tau_{i}^{N}\right)+P_{i}^{N}\tau_{i}^{I}\left(1-\tau_{i}^{R}\right)+P_{i}^{I}\left(1-\tau_{i}^{R}\right) \end{array}$$ (20)

According to Equation 20, in the steady state, $P_{i}^{I}=\varepsilon_{i}\ll 1$ $\tau_{i}^{N},\tau_{i}^{I},P_{i}^{N}$ are all small quantities. Simplifying Equation (20) yields a new equation, as shown in Equation 21:


$$\begin{array}{l} P_{i}^{I}=P_{i}^{S}\tau_{i}^{I}+P_{i}^{I}\left(1-\tau_{i}^{R}\right) \end{array}$$ (21)

Substituting $P_{i}^{I}=\varepsilon_{i}\ll 1$ into Equation 21 yields the following equation, as shown in Equation 22:


$$\begin{array}{l} \varepsilon_{i}=P_{i}^{S}\tau_{i}^{I}+\varepsilon_{i}\left(1-\tau_{i}^{R}\right) \end{array}$$ (22)

$\tau_{i}^{I}=\omega \alpha \left(P^{I}\right)\cdot n_{v_{i}}\cdot \underset{j}{\sum }rf\left(i_{3},j\right)P_{ij}^{I}\left(t\right)=\omega \alpha \left(P^{I}\right)\cdot n_{v_{i}}\cdot \underset{j}{\sum }rf\left(i_{3},j\right)\varepsilon_{j},$ Substituting $P_{i}^{S}=1-\varepsilon_{i}-\delta_{i},\;\tau_{i}^{R}=r$

into Equation 22 and noting that it can be further calculated as shown in Equation 23:


$$\begin{array}{l} \varepsilon_{i}=\omega \alpha \left(P^{I}\right)\cdot n_{v_{i}}\cdot \underset{j}{\sum }rf\left(i_{i},j\right)\varepsilon_{j} \end{array}$$ (23)

Equation 23 can be further simplified as shown in Equation 24:


$$\begin{array}{l} \frac{\mu }{\omega \alpha \left(P^{I}\right)-n_{v_{j}}}\varepsilon_{i}=\underset{j}{\sum }rf\left(i_{i},j\right)\varepsilon_{j} \end{array}$$ (24)

Equation 24 can be further simplified as shown in Equation 25:


$$\begin{array}{l} \underset{j}{\sum }\left[rf\left(i_{i},j\right)-\frac{\mu }{\omega \alpha \left(P^{I}\right)\cdot n_{v_{i}}}\sigma_{ij}\right]\varepsilon_{j}=0 \end{array}$$ (25)

In the equation, σ_*ij*_ is the Kronecker delta function. The necessary and sufficient condition for the system of Equations 25 to have a non-zero solution is given by Equation 26:


$$\begin{array}{l} \frac{\mu }{\omega^{I}\alpha \left(P^{I}\right)\cdot n_{y_{i}}}=\Lambda_{\max}\left(H\right) \end{array}$$ (26)

In the equation, *H* = (*h*_*ij*_), *h*_*ij*_ = *rf*(*i, j*), is an element of matrix *H*. Thus, the propagation threshold of rumor information I is given by Equation 27:


$$\begin{array}{l} \omega^{I}=\frac{\mu }{\Lambda_{\max}\left(H\right)\alpha \left(P^{I}\right)\cdot n_{v_{i}}} \end{array}$$ (27)

## 4 System simulation and results analysis

### 4.1 Analysis of propagation dynamics in different models

Small-world networks and scale-free network models can accurately describe and explain the structural and behavioral characteristics of real online social networks from different perspectives (24, 25). Compared with small-world networks and scale-free networks, hyperedges in hypernetworks based on hypergraphs can contain any number of nodes. Based on the small-world model, the scale-free model, and online social hypernetworks, this chapter verifies the rationality and effectiveness of the H-SNIR model under different network structures. It investigates the influence of user influence, topic popularity, and user interactions on rumor propagation. To eliminate the randomness of experimental results, each set of experiments in our study was independently simulated 500 times under the same initial conditions, and the average value was taken.

To validate the effectiveness and feasibility of the H-SNIR rumor propagation model constructed using the Markov chain method, this chapter also introduces Monte Carlo (MC) simulation and compares the evolution results of the two methods under identical parameter settings (11, 26). The experimental results in Figure 5 show that the simulation results of the Markov chain method and the Monte Carlo method maintain a largely consistent trend, demonstrating the reliability of the model. The Monte Carlo method primarily relies on computer-generated random numbers to simulate the rumor propagation process, thus introducing randomness. However, this randomness decreases as the number of simulation experiments increases. The Markov chain method calculates the proportions of users in different states based on the transition probabilities between states, and these proportions change only with variations in state transition probabilities. Since the Markov chain method overlooks randomness, it is reasonable for the proportions of users in the four states to be slightly larger or smaller than those obtained by the Monte Carlo method.

**Figure 6:**
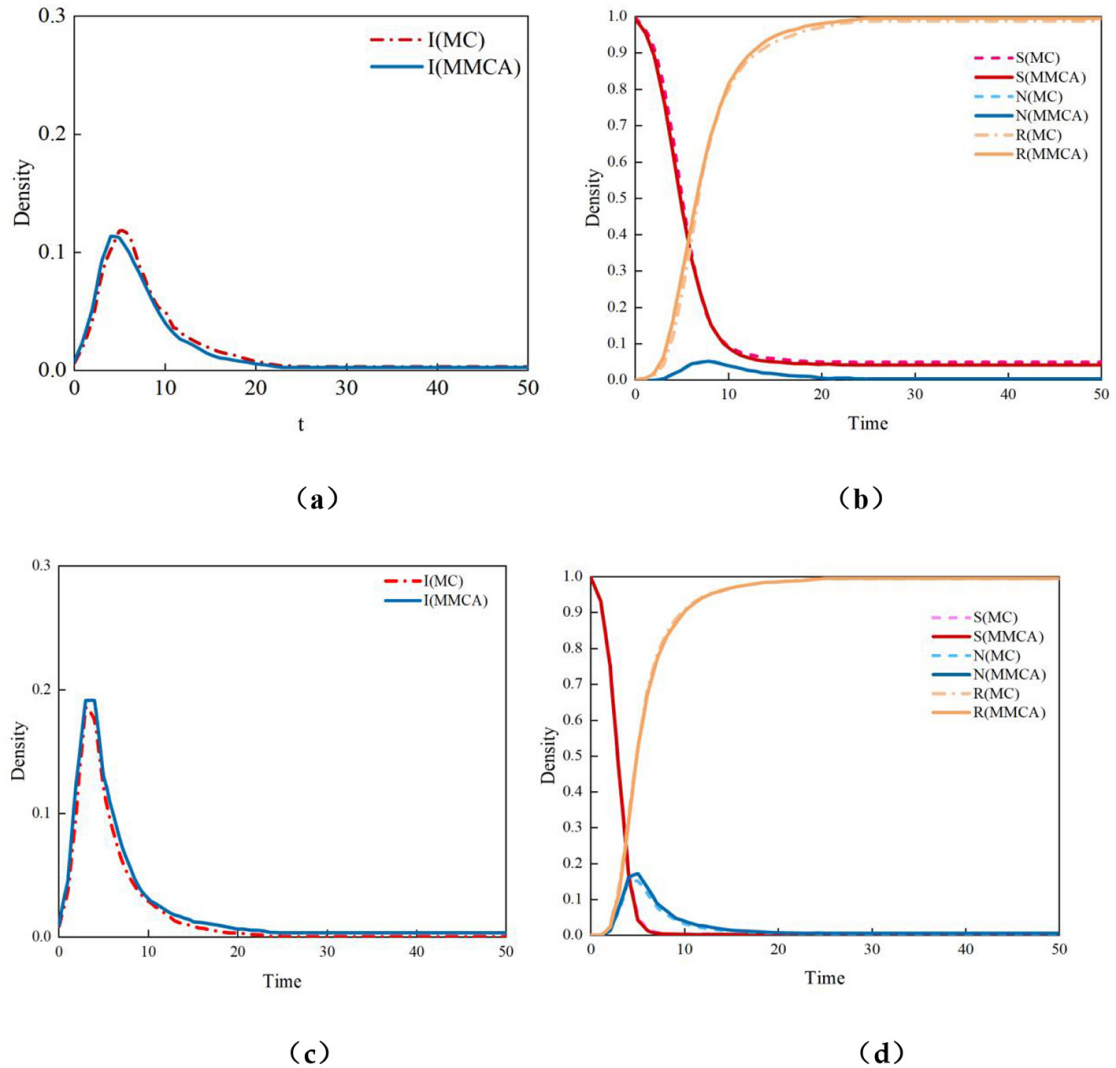
Density curves of four states based on the MC and MMCA methods: **(a)** Density curve of state I when λ = 0.1, **(b)** Density curves of states S, N, and R when λ = 0.1, **(c)** Density curve of state I when λ = 0.2, **(d)** Density curves of states S, N, and R when λ = 0.2.

The experimental results in Figure 6 demonstrate that the simulation outcomes from both methods exhibit a fundamentally consistent trend. Since the time complexity of the Markov chain simulation is O(N^2^), whereas that of the Monte Carlo simulation is O(N), the latter has lower time complexity. Therefore, the Monte Carlo method will be employed for subsequent experimental simulations.

**Figure 7:**
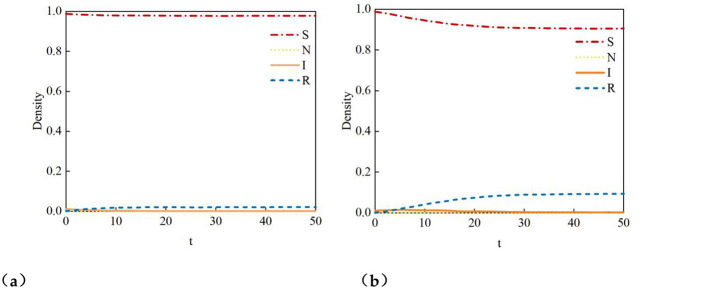
Density curves of four states. **(a)** Density curves of four states when λ = 0.01, **(b)** Density curves of four states when λ = 0.03.

The Monte Carlo simulation is used to verify Theorem 1. With parameters μ1 = μ_2_ = 0.3 set, the calculated result of Theorem 1 shows that the propagation threshold for infected nodes I is $\lambda_{c}^{I}$ = 0.0216. The simulation results are illustrated in Figure 6. From Figure 7a, it can be observed that when the propagation rate λ1 = 0.01 < $\lambda_{c}^{I}$ = 0.0216, the proportion of infected nodes I is close to zero, and the proportion of immunized nodes (i.e., users in state R) is nearly zero. The system state remains below the propagation threshold, indicating that rumors fail to spread effectively. However, as shown in Figure 7b, when the propagation rate λ1 = 0.03 >$\lambda_{c}^{I}$ = 0.0216, the proportion of infected nodes I is slightly above zero, and the proportion of immunized nodes is approximately 0.1. When the propagation rate exceeds the propagation threshold, rumors exhibit sustained.

**Figure 8:**
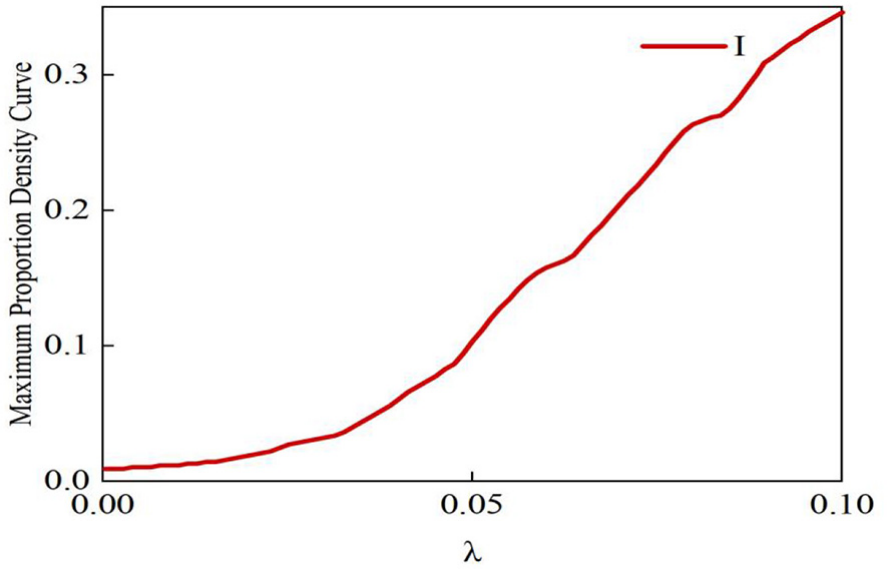
Maximum density curve of information I when λ = 0.

Figure 8 shows that the maximum proportion of information I propagation increases with the rise in the propagation rate λ1. When λ1 exceeds the propagation threshold, the social network exhibits a clear phenomenon of rumor propagation, and the scale of rumor spread continues to expand as λ*1* increases. Additionally, the propagation threshold of information I is approximately 0.02, which aligns closely with the calculated propagation threshold $\lambda_{c}^{I}$ = 0.0216 from Theorem 1. This consistency not only validates the accuracy of Theorem 1 but also demonstrates that the Monte Carlo simulation can effectively capture the phase transition process of rumor propagation.

To validate the rationality and effectiveness of the H-SNIR model, simulation experiments were conducted based on a hypernetwork structure with 590 nodes, and a comparative analysis was performed among the SIS, SIR, and H-SNIR models. A comparison of these models is presented in Table 1. At the beginning of the experiment, a node was randomly selected as the initial infected (I) node, while all other nodes were in the susceptible (S) state. In the SIS model, the infection probability and recovery probability were set to 0.3 and 0.7, respectively. In the SIR model, the infection probability and immunization probability were set to 0.3 and 0.7, respectively. In the H-SNIR model, the transition probabilities were as follows: S to N: 0.3, S to I: 0.7, N to I: 0.7, N to R: 0.3, I to R: 0.3. Figure 8 illustrates the density curves of users in different states for the SIS, SIR, and H-SNIR models.

**Table 1:** Comparison of different models.

Model	Model description	Model analysis
**SIS**	The users are divided into two categories: susceptible state S and infected state I. The propagation rule is “Susceptible state-Infected state -Susceptible state”	In online social networks, users who are actively spreading information are unlikely to forget the information they spread in a short period of time. Therefore, using the SIS model to simulate real networks is overly idealized
**SIR**	The users are divided into three categories: susceptible state S, infected state I, and recovered state R. The propagation rule is “Susceptible state-Infected state- Recovered state”	The SIR model describes user propagation behavior as: users forward information when interested and ignore it when uninterested
**H-SNIR**	Users are divided into four categories: Susceptible state (S), Exposed state (E), Infected state (I), and Recovered state (R). The propagation pattern follows “Susceptible state-Exposed state -Infected state-Recovered state” (S-E-I-R)	Based on the propagation behavior of users in online social networks after receiving information, we have defined the propagation rules of the H-SNIR model. In the process of user state transitions, the influence of both user-specific factors and information attributes on information dissemination is taken into account

**Figure 9:**
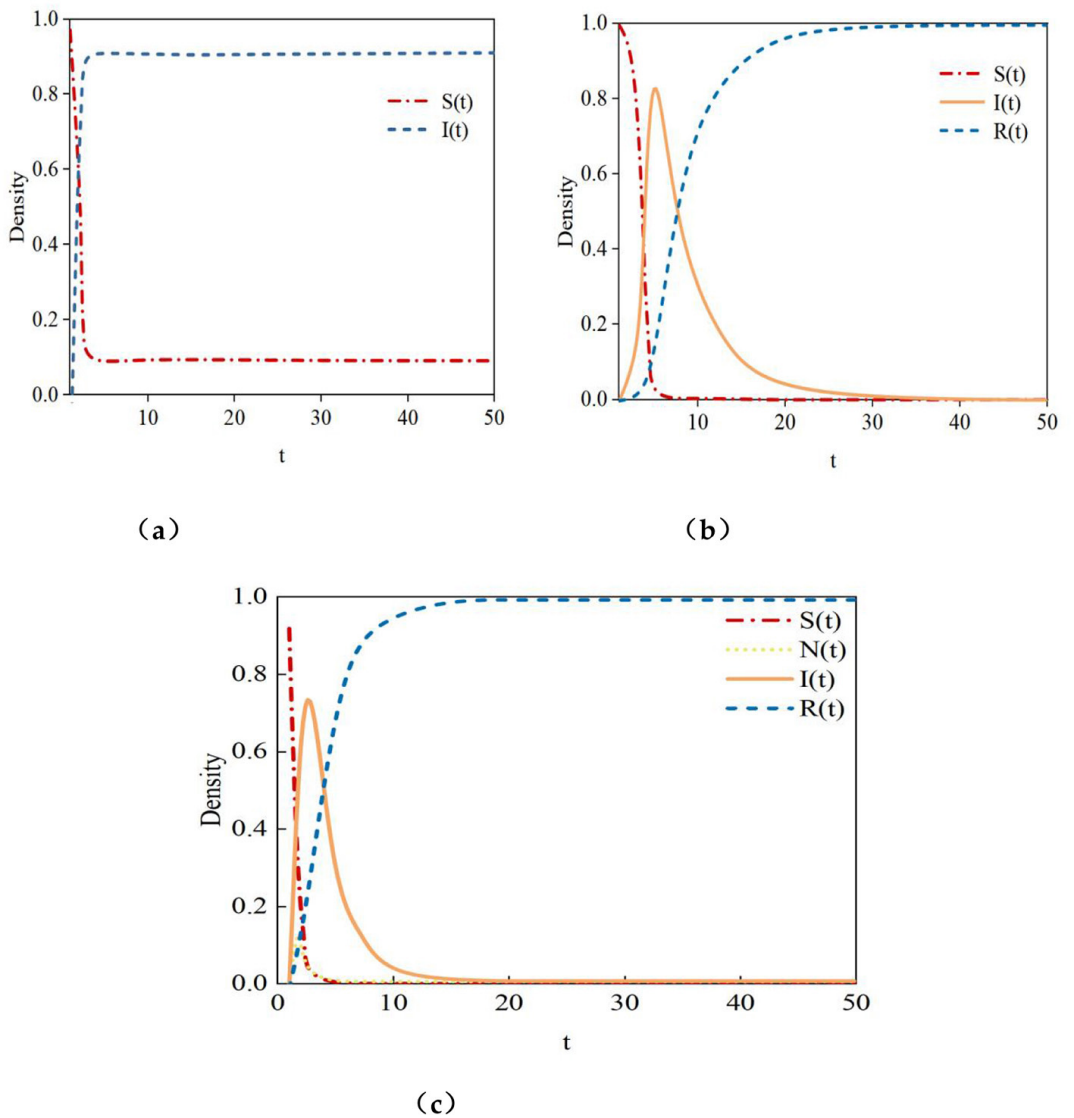
H-SNIR model and other models. **(a)** SIS model, **(b)** SIR model, **(c)** H-SNIR model.

The Monte Carlo simulation results are shown in Figure 9. The experimental results in Figure 9a indicate that in the SIS model, approximately 90% of users remain in the infected (I) state, which does not align with the real-world propagation patterns of rumors. Assuming that individuals, after recovering from infection, can be reinfected by rumor information, a scenario where a large proportion of users remain in the infected state for an extended period suggests an absence of effective factors to curb the spread of rumors in the propagation process. This contradicts the actual dynamics of rumor propagation. Users in online social networks are diverse, and their reactions to rumor information vary. Multiple factors exist to suppress the spread of rumors, such as some users having strong discernment abilities and not easily propagating rumors. Therefore, it is unlikely that over 90% of users would continuously spread rumor information.

The experimental results in Figure 9b show that in the SIR model, rumor information spreads rapidly during the initial stage, entering an outbreak phase directly. This model divides the population into susceptible (S), infected (I), and recovered (R) nodes. Once rumor information begins to spread, the initial presence of a large number of susceptible nodes allows the rumor to quickly diffuse into an outbreak phase. However, in real social networks, rumor propagation is not so straightforward. The spread of rumors is constrained by various factors, and not all users will accept and propagate rumors upon receiving them. For example, the dissemination of rumors may require time, and the strength of social ties between users can affect the speed of rumor transmission. Factors such as the novelty of the topic and user influence also impact whether social users choose to spread rumors. Therefore, the assumption of an immediate outbreak in the SIR model is overly idealized.

The H-SNIR model proposed in this paper introduces hesitant nodes, better reflecting the diversity of social network users. Susceptible nodes, upon receiving information, may not only become infected but also enter a hesitant state, temporarily undecided about whether to forward the rumor. Additionally, the constructed rumor propagation model in this chapter considers factors such as topic popularity, user influence, and user interactions on rumor spread. The simulation results in Figure 9c show that after susceptible nodes encounter rumor information, most transform into infected nodes, while some become hesitant nodes. As rumors spread across the network, hesitant nodes may either propagate or refrain from spreading the rumor. This aligns more closely with the dynamics of rumor propagation in real social networks.

### 4.2 The influence of different network structures on the spread of online rumors

The study explores the dynamic processes and patterns of rumor propagation under different network structures, selecting the node with the highest degree in the network as the initial propagation node (if multiple nodes have the same degree, one is chosen randomly). In real social networks, the number of connections (degree) varies among users, and the user base is vast. In this chapter, the maximum degree in the small-world network is set to 16, with a node count of 923; in the scale-free network, the maximum degree is set to 34, with a node count of 241; and in the hypernetwork, the maximum hyperdegree is set to 6, with a node count of 590. Other parameter settings are as shown in Table 2.

**Table 2:** Simulation parameter values.

α	β	η	ξ	λ
0.3	0.7	0.7	0.3	0.3

**Figure 10:**
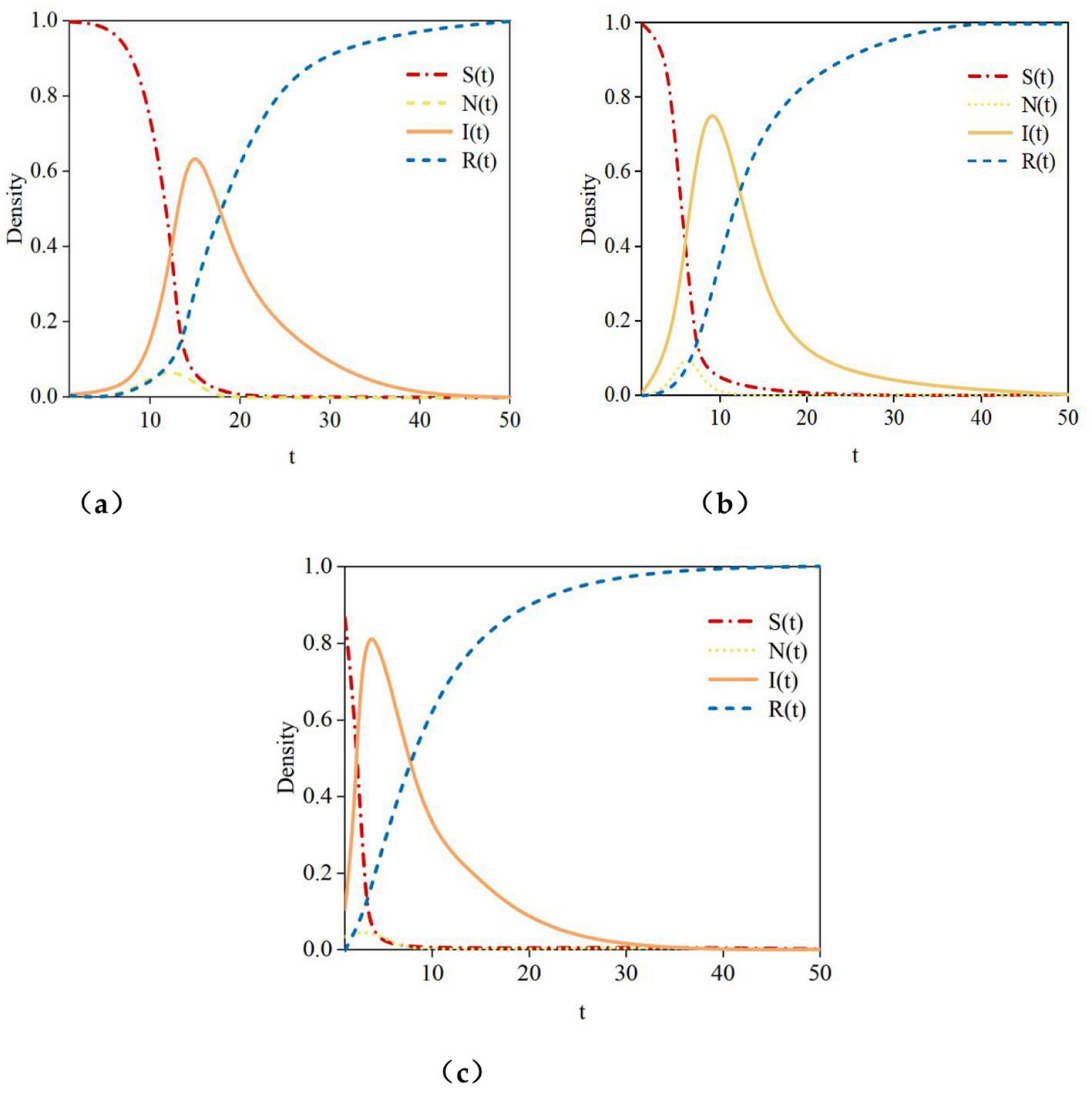
Rumor propagation curves under different network structures. **(a)** small-world network, **(b)** scale-free network, **(c)** Hypernetwork.

**Figure 11:**
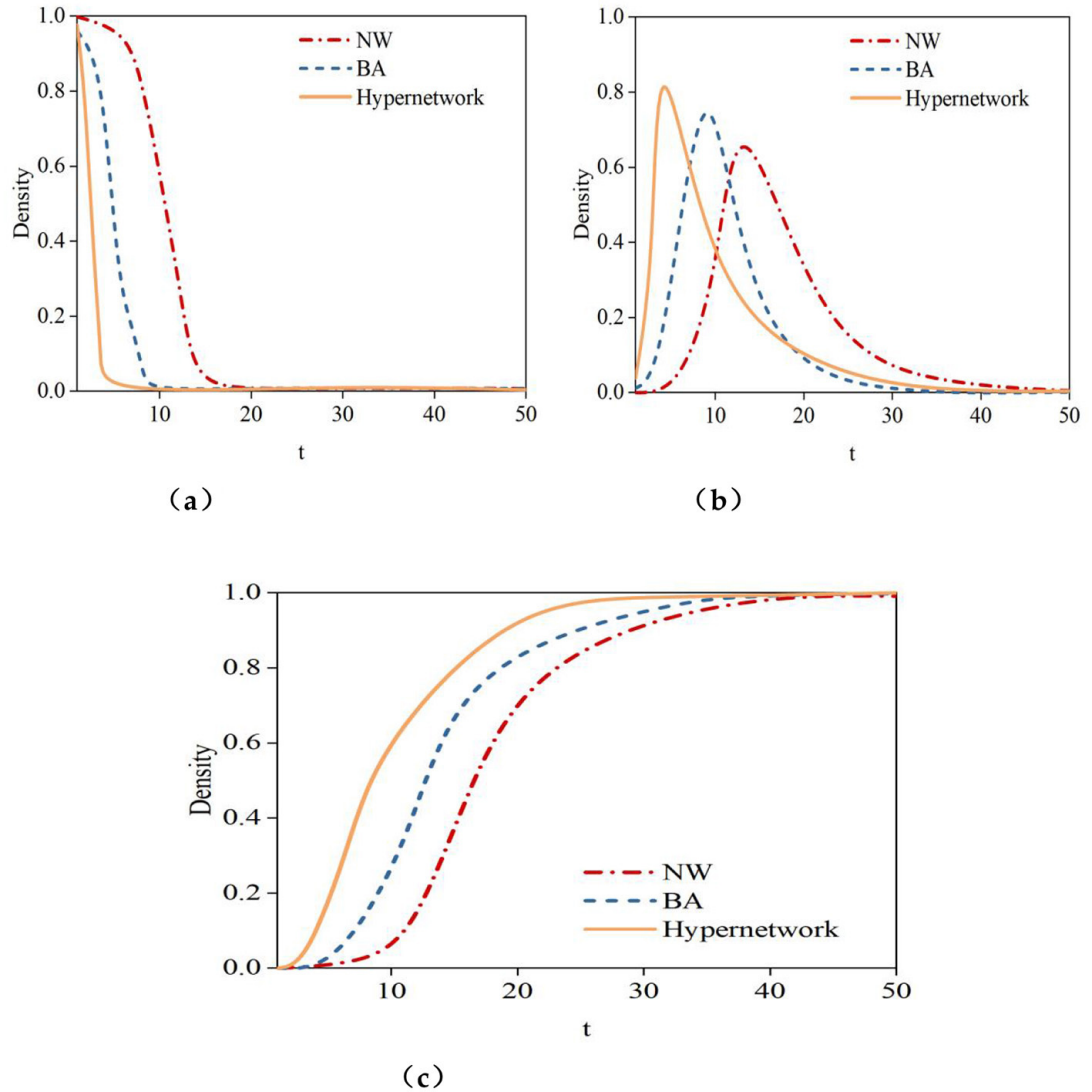
The changes in density of nodes in different states within the network. **(a)** Unknown node (S), **(b)** Infected node (I), **(c)** Immune node (R).

Figure 10 illustrates the density curves over time of susceptible nodes **(S)**, hesitant nodes **(N)**, infected nodes **(I)**, and recovered nodes **(R)** in small-world networks, scale-free networks, and hypernetworks. Figure 11 indicates that, across the three network structures, the density trends of nodes in different states over time are similar. The density curve of susceptible nodes **(S)** shows a rapid decline until it reaches zero. The density curve of hesitant nodes **(N)** rises quickly in the initial stage, peaks, and then declines rapidly to zero. In the early stage of rumor propagation, most users can receive the information. However, some users do not immediately spread the rumor, causing the density curve of hesitant nodes **(N)** to rise rapidly to its peak. After reaching the peak, the majority of users have already received the information and gradually begin either spreading it or refusing to do so, leading to a decline in the density curve of hesitant nodes **(N)** until it reaches zero. The density curve of infected nodes **(I)** first rises to a peak and then declines. This occurs because susceptible nodes receive and spread the rumor, while hesitant nodes also begin to propagate it, causing the density curve of infected nodes **(I)** to rise quickly to its peak. After the peak, infected nodes gradually refuse to spread the rumor or lose the ability to do so, resulting in a downward trend in the density curve of infected nodes **(I)**. The density curve of recovered nodes **(R)** increases rapidly at first, and as nodes in other states transition to the recovered state, the density of recovered nodes tends toward 1. Although the propagation trends of rumor information in the three network structures exhibit similar patterns, there are also notable differences. To more clearly observe the propagation trends of rumor information in these three network structures, Figure 10 presents the density curves over time for susceptible nodes (S), infected nodes (I), and recovered nodes (R) across the three network types.

The experimental results in Figure 10 indicate:

(1) The time required for susceptible nodes (S) to decline to their minimum varies. In small-world networks, scale-free networks, and hypernetworks, susceptible nodes (S) reach their minimum at *t* = 18, *t* = 9, and *t* = 4, respectively. The hypernetwork achieves this minimum in the shortest time compared to scale-free and small-world networks, suggesting that rumor information spreads fastest in online social hypernetworks.
(2) The time required for infected nodes (I) to peak differs, as does the peak value itself. In small-world networks, scale-free networks, and hypernetworks, infected nodes (I) peak at *t*=18, *t* = 11, and *t* = 6, with peak values of 0.62, 0.72, and 0.81, respectively. The hypernetwork not only reaches its peak in the shortest time but also exhibits the highest peak density of infected nodes (I). This demonstrates that rumor information spreads fastest and reaches the widest range in hypernetworks. This is because, in small-world and scale-free networks, edges typically connect only two nodes, whereas hypernetworks use hyperedges to connect any number of nodes, more accurately describing the complex relationships in online social networks. This structural feature enables rumors to spread rapidly in hypernetworks, leading to a quicker rise in the number of infected nodes and a higher peak density.
(3) The time required for recovered nodes (R) to reach a steady state varies, as does the steady-state value. In small-world networks, scale-free networks, and hypernetworks, recovered nodes (R) achieve steady states at *t* = 40, *t* = 37, and *t* = 28, respectively. The recovered state represents the final outcome of information propagation. The time required to reach a steady state reflects the speed of information spread, while the steady-state value indicates the scale of propagation. A shorter time to reach steady state and a larger steady-state value correspond to faster and broader rumor propagation.

### 4.3 Influence of user and information attributes on the spread of online rumors

In online social networks, information can spread on a large scale, and influencing factors across different dimensions play a key role in the propagation of rumors. This chapter validates the impact of three influencing factors—topic popularity, user influence, and user interactions—on the mechanisms of online rumor propagation.

(1) **User Influence:** In the study, nodes with the smallest influence α(*P*_*I*_) = 0.1, moderate influence α(*P*_*I*_) = 0.3, and the largest influence α(*P*_*I*_) = 0.5 in the hypernetwork were selected as initial infected nodes (if users had the same influence, selection was randomized) to analyze the impact of user influence on rumor propagation. Figure 11 shows the density curves of susceptible nodes (S), hesitant nodes (N), infected nodes (I), and recovered nodes (R) over time under different levels of initial infected node influence.

**Figure 12:**
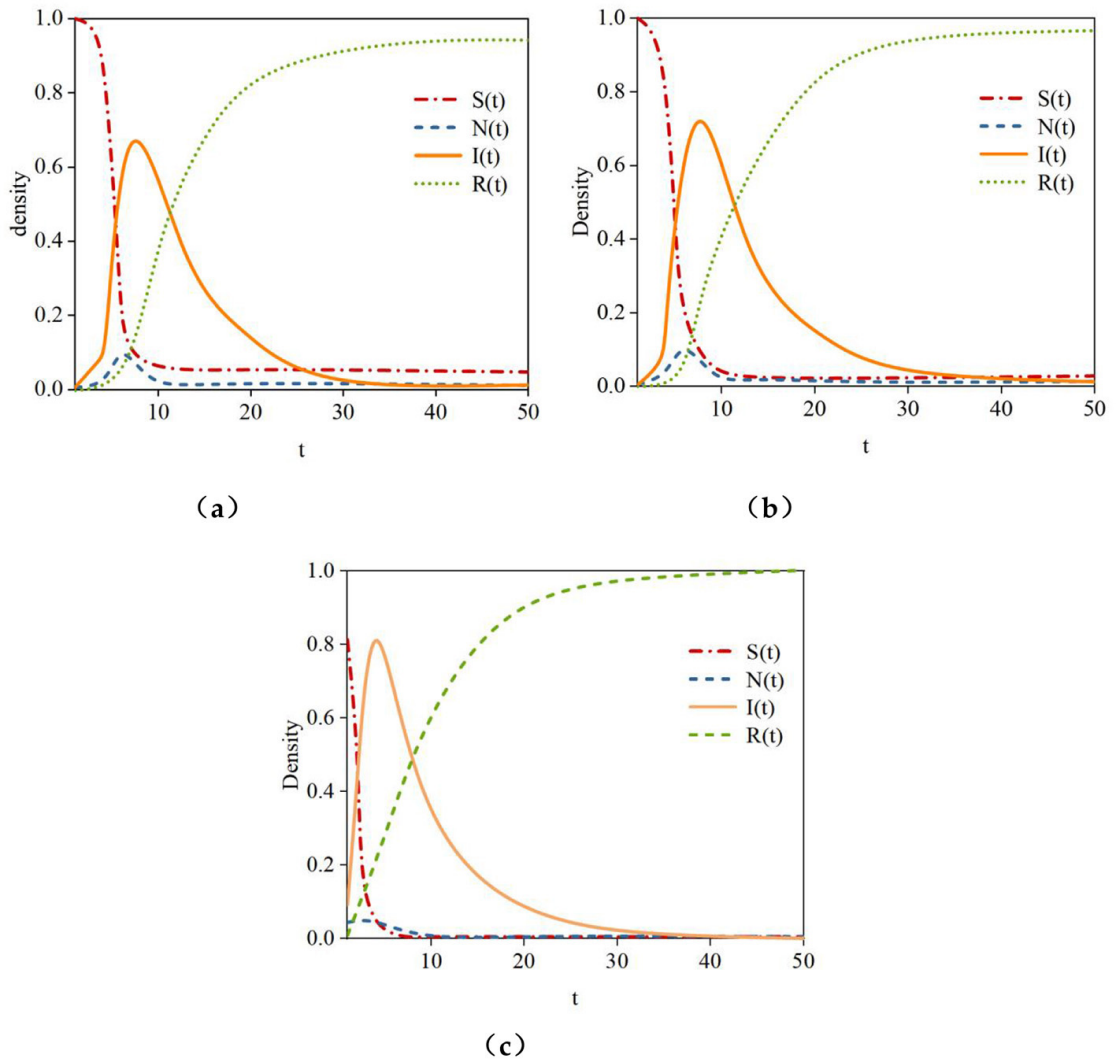
Density variation of different node types under the influence of varying user influence levels. **(a)** α (*P*^*I*^) = 0.1, **(b)** α (*P*^*I*^) = 0.3, **(c)** α (*P*^*I*^) = 0.5.

The simulation results in Figure 12 indicate that as the influence of the initial infected node gradually increases, the time for susceptible nodes (S) to reach a steady state is *t* = 9, *t* = 8, and *t* = 5, respectively, with their density gradually decreasing to 0. The time for hesitant nodes (N) to reach their peak is *t* = 5.5, and *t* = 3, respectively, and the steady-state density of hesitant nodes gradually decreases to 0.9, 0.8, and 0.2. The time for infected nodes (I) to reach their peak gradually decreases to *t* = 9, *t* = 8.5, and *t* = 6.5, with peak densities of 0.68, 0.75, and 0.82, respectively. The time for recovered nodes (R) to reach a steady state is *t* = 40, *t* = 38, and *t* = 30, with steady-state densities of 0.92, 0.96, and 1, respectively. This shows that as user influence increases, the time for all types of nodes to reach a steady state shortens. Additionally, higher influence enhances trust among users, reducing the number of hesitant nodes and encouraging more nodes to believe and spread the rumor.

(2) **Topic Popularity**: To analyze the impact of topic popularity on rumor propagation, the popularity value of the rumor *n*_*vi*_ was set to 0.1, 0.3, and 0.5. Figure 12 shows the density curves of susceptible nodes (S), hesitant nodes (N), infected nodes (I), and recovered nodes (R) over time in the hypernetwork under these three popularity settings.

**Figure 13:**
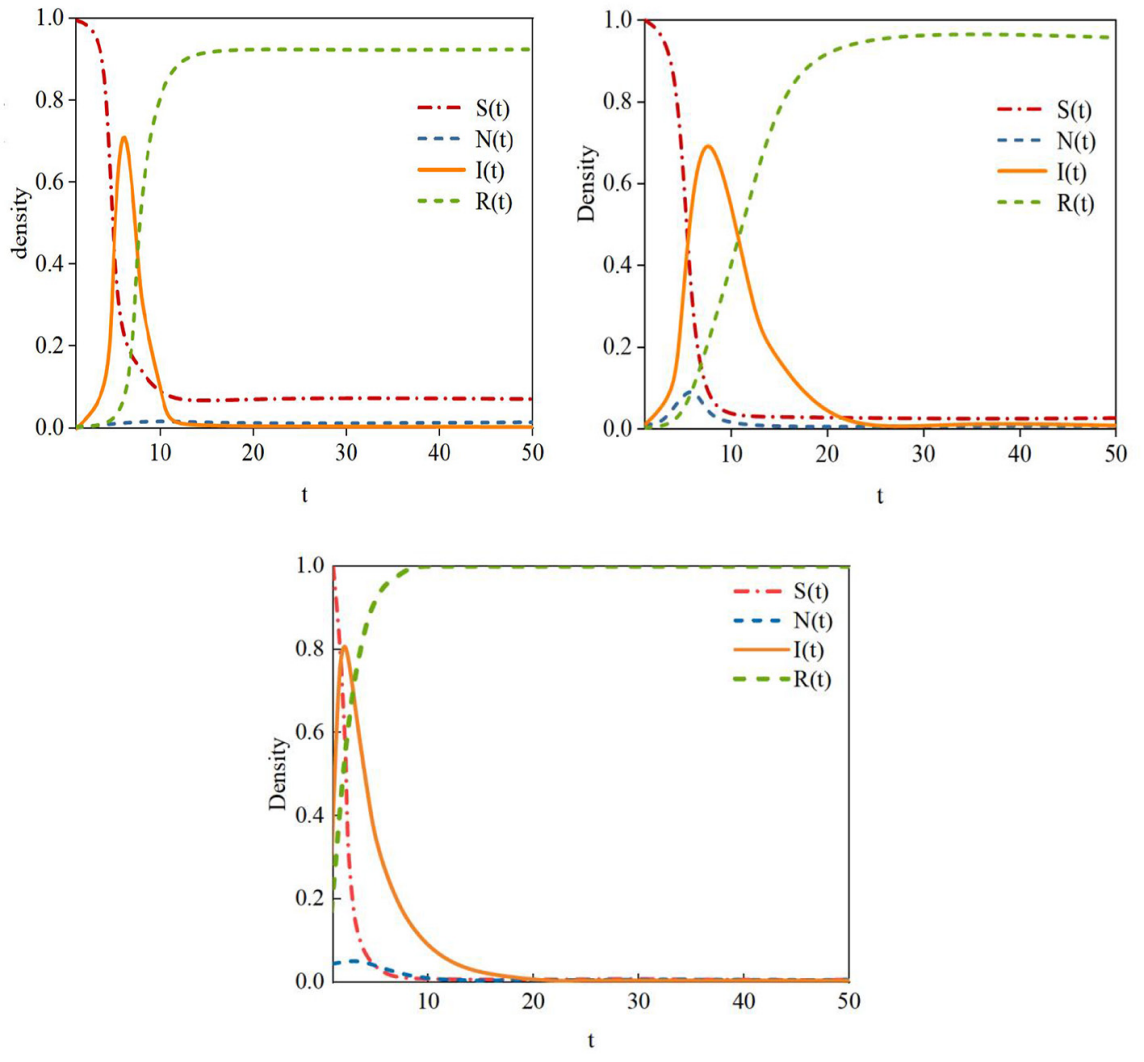
*n*_*vi*_= 0.1, *n*_*vi*_= 0.3, and *n*_*vi*_= 0.5 curves of density variation for different node types.

The simulation results in Figure 13 indicate that topic popularity significantly affects both the speed and scope of rumor propagation. As the topic popularity value *n*_*vi*_ gradually increases, the time for susceptible nodes (S) to reach a steady state is *t* = 8, *t* = 6, and *t* = 3, with steady-state densities of 0.03, 0.02, and 0, respectively. The time for hesitant nodes to reach their peak is *t* = 5.5, *t* = 3.5, and *t* = 3, with peak densities of 0.09, 0.07, and 0.03, respectively. The time for infected nodes to reach their peak density is *t* = 6, *t* = 5.5, and *t* = 4, with peak densities of 0.68, 0.7, and 0.81, respectively. The time for recovered nodes (R) to reach a steady state is *t* = 22, *t* = 15, and *t* = 9, with steady-state densities of 0.92, 0.95, and 1, respectively. Higher topic popularity accelerates rumor propagation, as susceptible and hesitant nodes are more likely to convert to infected nodes, increasing the number of infected nodes. However, as topic popularity increases, some active rumor spreaders cease propagation, leading to an increase in the number of recovered nodes.

**Figure 14:**
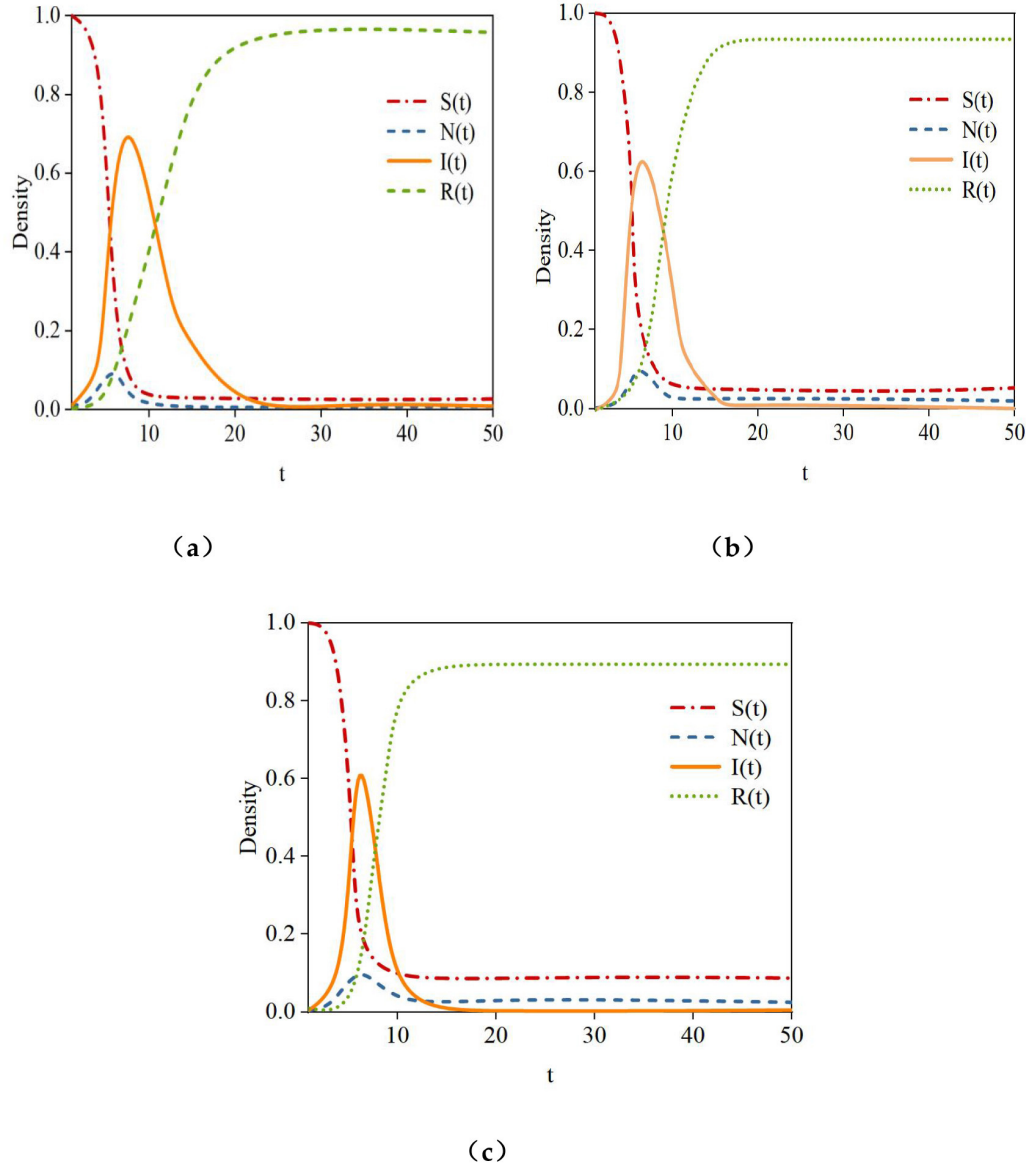
Node density variation curves under the influence of user interactions. **(a)** *rf*_(i, j)_ = 0.1, **(b)** *rf*_(i, j)_ = 0.0.3, **(c)** *rf*_(i, j)_ = 0.5.

(3) Interactions Among Users: The study sets the interaction strength among users, denoted as *rf*_(i, j)_, to 0.1, 0.3, and 0.5, respectively, to examine the patterns of rumor propagation under varying levels of user interaction. The simulation results in Figure 14 demonstrate that in the hypernetwork, the densities of susceptible nodes (S), hesitant nodes (N), infected nodes (I), and recovered nodes (R) vary depending on the strength of interactions among users.

During the rumor propagation process, as the strength of interactions among users gradually increases: The time for susceptible nodes (S) to reach a steady state is *t* = 13, *t* = 12, and *t* = 10.5, with steady-state densities of 0.09, 0.05, and 0.01, respectively. The time for hesitant nodes (N) to reach their peak is *t* = 6, *t* = 5.5, and *t* = 3, with peak densities of 0.09, 0.08, and 0.06, respectively. The time for infected nodes (I) to reach their peak is *t* = 8, *t* = 7, and *t* = 4.5, with peak densities of 0.61, 0.63, and 0.68, respectively. The time for recovered nodes (R) to reach their peak is *t* = 17, *t* = 15, and *t* = 12, with peak densities of 0.91, 0.93, and 0.98, respectively. In online social networks, interactions among users facilitate the exchange of rumor information, enhancing the alignment between the popularity and content of rumors and the interests of neighboring users within social groups. As a result, the speed of information propagation accelerates, and its diffusion scope expands.

## 5 Conclusions and suggestions

During public crisis events, rumor information can spread more widely and rapidly through social groups. Given the uniqueness and complexity of rumor dissemination in online social networks, conducting in-depth research into the dynamic processes of rumor propagation within hypernetworks becomes critically important.

(1) To address the limitation of previous studies that only focused on the impact of network structure on the path of rumor propagation, our study constructs a novel H-SNIR rumor propagation model based on a hypernetwork that incorporates a neglected mechanism. This model provides an in-depth analysis of the influence of topic popularity, user influence, and user interaction on rumor forwarding behavior. By quantitatively representing these three-dimensional influencing factors, the model quantitatively evaluates the contribution of different factors to online rumor propagation during sudden public crisis events. It transforms the fixed transition probabilities between nodes into computable dynamic parameters, enabling a scientific understanding of the propagation mechanism of online rumors and constructing a comprehensive cognitive system for the laws governing rumor propagation.
(2) To more accurately calculate the impact of different influencing factors on rumor information dissemination, this study incorporates Markov Chain (MMCA) theory to characterize the dynamic changes between different system states. Based on these influencing factors, it accurately calculates the transition probabilities of nodes between different states, abstracting the rumor propagation process into a probabilistic system of state transitions. This precisely characterizes the dynamic process of the rumor propagation mechanism and more realistically simulates the complex and non-steady-state propagation behaviors in real-world social networks.

## Data Availability

The original contributions presented in the study are included in the article/supplementary material, further inquiries can be directed to the corresponding authors.
